# Heat inactivation of stable proteinaceous particles for future sample return mission architecture

**DOI:** 10.3389/fmicb.2022.911091

**Published:** 2022-08-09

**Authors:** Emily P. Seto, Aspen L. Hirsch, Wayne W. Schubert, Pavithra Chandramowlishwaran, Yury O. Chernoff

**Affiliations:** ^1^Honeybee Robotics, Altadena, CA, United States; ^2^Biotechnology and Planetary Protection Group, Jet Propulsion Laboratory, California Institute of Technology, Pasadena, CA, United States; ^3^School of Biological Sciences, Georgia Institute of Technology, Atlanta, GA, United States

**Keywords:** backwards planetary protection, planetary protection, mars sample return, prion inactivation, prions, biological indicator, sample return mission

## Abstract

The National Aeronautics and Space Administration (NASA) and the European Space Agency (ESA) are studying how to improve the safety of future planetary science sample return missions that would bring back materials to Earth. Backward planetary protection requirements have been identified as a critical technology development focus in order to reduce the possibility of harm to Earth’s biosphere from such returned materials. In order to meet these challenges, NASA has identified the need for an appropriate suite of biological indicators (BIs) that would be used to develop, test, and ultimately validate sample return mission sterilization systems. Traditionally, BIs are defined as test systems composed of viable microorganisms that are inactivated when necessary conditions are met during sterilization procedures, providing a level of confidence in the process. BIs used traditionally at NASA have been driven by past mission requirements, mainly focused on spore-formers. However, spore-based BIs are insufficient as the only analog for a nominal case in sample return missions. NASA has directed sample return missions from habitable worlds to manage “potential extraterrestrial life and bioactive molecules” which requires investigation of a range of potential BIs. Thus, it is important to develop a mitigation strategy that addresses various known forms of biology, from complex organisms to biomolecular assemblies (including self-perpetuating non-nucleic acid containing structures). The current effort seeks to establish a BI that would address a stable biomolecule capable of replication. Additional engineering areas that may benefit from this information include applications of brazing, sealing, and impact heating, and atmospheric entry heating. Yeast aggregating proteins exhibit aggregation behavior similar to mammalian prion protein and have been successfully employed by researchers to understand fundamental prion properties such as aggregation and self-propagation. Despite also being termed “prions,” yeast proteins are not hazardous to humans and can be used as a cost effective and safer alternative to mammalian prions. We have shown that inactivation by dry heat is feasible for the prion formed by the yeast Sup35NM protein, although at higher temperature than for bacterial spores.

## Introduction

As upcoming missions seek to understand life beyond our planetary body, sample return missions will require development of a mitigation strategy to address any potential hazard to Earth’s biosphere. As signatories of the 1967 Outer Space Treaty, the United States has agreed to the practice of Planetary Protection (PP) which entails protecting solar system bodies from “forward contamination” by Earth life and protecting Earth from “backward contamination” returned from other solar system bodies (NASA, 2008; NPD 8020.7G). The Committee on Space Research (COSPAR) established by the International Council for Science in 1958 further outlines an international planetary protection policy (Kminek et al., 2017; Committee on Space Research, 2020). This planetary protection policy includes categorization of space missions according to the type of encounter (i.e., flyby, orbiter, lander or sample return) and the target (Kminek et al., 2017; NASA Science Mission Directorate, 2017). Sample return missions are classified as PP Category V, with details that depend on the target planetary body from where samples will be collected. Within this category, missions are classified as the following: “unrestricted” if samples are returned from solar system bodies that have no indication of indigenous life forms or “restricted” if there is scientific evidence to support potential indigenous biological life (NPR 8715.24; NASA, 2021; Committee on Space Research, 2020). As part of the effort to develop strategies for meeting stringent Category V requirements, we investigate backward planetary protection strategies developed to reduce the risk of harmful contamination of Earth with uncontained returned samples. To meet these challenges, the development and implementation of a suitable strategy for the sterilization of returning material is necessary.

The scientific consensus from prior sample return working groups, reviews, and other meetings is that the existing biological indicators typically used to assess sterilization systems are not sufficient to adequately assess the efficacy of a sample return sterilization approach for backward-contamination (Craven et al., 2020). Additional engineering areas that may benefit from this information include applications of brazing, sealing, and impact heating and atmospheric entry heating. Given that heat-resistant spores have been tested for multiple backward PP-related experiments, a combination of the strategies with our approach will be considered to understand and reduce the viability of potentially uncontained returned material through the entire mission architecture.

Traditional self-contained biological indicators (ISO 11138-7, 2019) employ test systems composed of viable microorganisms that are inactivated when necessary conditions are met during sterilization procedures, providing a level of confidence in the process (Sandle, 2019). They are somewhat limited by past mission requirements, which mainly focused on spore-forming bacteria. Unfortunately, it has become clear that spore-based biological indicators are insufficient for a nominal case for both restricted and unrestricted sample return missions (Craven et al., 2020). It was suggested that use of yeast prions could be used as an analog for the bioactive molecules (Craven et al., 2020). Currently, heat resistant biological indicators used by NASA include *Bacillus atrophaeus* ATCC 9372 often used for dry heat microbial reduction (DHMR) processing and an industry standard biological indicator species for dry-heat sterilization (McDonnell, 2017; Bancroft, 2020). For the PP NASA standard assay, *B. atrophaeus* ATCC 9372 has also been used to as a biological indicator for cleanroom-associated surfaces bioburden assessment. *Bacillus* sp. ATCC 29669 is another spore-former associated with NASA flight assembly cleanrooms. The dry heat-resistance of *Bacillus* sp. ATCC 29669 represents the heat-hardy fraction within the NASA heat lethality curves, that is approximately 20 to 35 times more resistant than of *B. atrophaeus*. This resistance is based on D-values which are defined at the decimal reduction time or the time required to reduce a population by 90% at a specified temperature. The inactivation method is known as Dry Heat Microbial Reduction (DHMR). *Bacillus* sp. ATCC 29669 continues to be used as a test organism to validate effects of high heat and DHMR. Many other living systems and their products that exist on Earth should be considered. Developing a mitigation strategy to address all potential life-related forms (based on life as we know it on Earth), including self-perpetuating biomolecules, will be important to address or alleviate concerns about bringing back potentially biologically active material. Prions are biological molecules with the name derived from “proteinaceous infectious particles” (Prusiner, 1982). Prions have proven to be highly resistant to traditional sterilization modalities such as ethylene oxide (EO) and gamma radiation that are usually sufficient for microbial inactivation (Colby and Prusiner, 2011).

At a molecular level, prions are infectious protein isoforms, typically composed of highly ordered fibrous aggregates (amyloids). A prion protein can convert from a soluble form of the same or similar protein into an amyloid form, that is protease-resistant and insoluble (Bruce, 2003; Colby and Prusiner, 2011). Mammalian prion PrP^Sc^ is an infectious agent causing fatal infectious disease such as Creutzfeldt-Jakob disease (CJD) in humans and bovine spongiform encephalopathy (BSE) in cattle (Prusiner, 1998; Colby and Prusiner, 2011). Although prions lack nucleic acid material such as DNA and RNA, they are capable of “Darwinian” evolution and transmission, undergoing “mutations” and “adaptive” changes (Li et al., 2010). High resistance of prions to traditional sterilization methods is due to their unique and stable highly ordered structure (Brown et al., 1990). Many biocidal treatments that are known to be detrimental to microorganisms (for example, DNA damaging treatments) are not effective against prions (McDonnell, 2013). Thus, anti-prion “sterilization” techniques should be based on different principles compared to microorganisms.

The Centers for Disease Control and Prevention (CDC) defines sterilization as a process that destroys or eliminates all forms of microbial life that is carried out by physical or chemical methods (Rutala and Weber, 2001; Rutala et al., 2008). Since this definition emphasizes microorganisms as living forms, the term “inactivation” would better describe the loss of prion’s ability to convert a substrate protein into a prion state (Giles et al., 2017). According to the World Health Organization (WHO), aggressive inactivation strategies such as chemical, physical, and mechanical methods that are usually satisfactory against microbes or viruses, have proven insufficient against PrP^Sc^ (WHO infection control guidelines, 1999). One technique for PrP^Sc^ inactivation that was validated is soaking material in sodium hydroxide (or sodium hypochlorite), removing it and then heat inactivation at 134°C (Rutala and Weber, 2001). However, the use of NaOH and other corrosive chemicals is not applicable for space mission needs due to risks associated during flight. The storage and application of such chemicals during a long duration robotic mission is complex, and was thus ruled out as an option. Therefore, there is a need to address the knowledge gap in prion inactivation.

Dry heat is an accepted process for sterilization not only in pharmaceutical industry but also in the aerospace community and for forward planetary protection efforts. It has been selected as the primary option for backward planetary protection sterilization strategies (Craven et al., 2020). Dry heat is also a NASA-approved technique for microbial reduction for forward planetary protection; therefore, there is extensive experience in reducing the bioburden carried by outward-bound hardware. It is compatible with spacecraft parts and can be applied in vacuum. Heat penetrates below surfaces and encapsulated bioburden and it will be crucial for validating the Mars sample return sterilization system. Heat has been shown to inactivate proteins by increasing kinetic energy of molecules, causing the materials to dissociate.

Studies of prion inactivation are complicated by the biosafety concerns related to prion pathogenicity. This could be overcome by employing prions of yeast *Saccharomyces cerevisiae* as surrogates for sterilization testing. Yeast contains a variety of proteins that can form amyloid-based prions (Liebman and Chernoff, 2012). These proteins are not homologous to mammalian PrP by sequence and are not harmful to humans, however they possess remarkable similarities to PrP^Sc^ and other mammalian amyloids in regard to principles of transmission and structural organization. Yeast prions form fibrous structures enriched in beta-sheets, exhibiting typical characteristics of amyloids, and are capable of converting monomeric protein into a prion form, as in the case of PrP^Sc^ (Chernoff et al., 2020). In yeast cells, prions cause phenotypically detectable changes heritable *via* cytoplasm (Liebman and Chernoff, 2012). Among more than 10 proteins proven to form a prion in yeast cells to date, the Sup35 protein is the most extensively studied (Liebman and Chernoff, 2012). The prion-forming domain of Sup35 is located in the N-proximal region and is distinct from the C-proximal region responsible for the cellular function of Sup35 (termination of translation). The middle (M) region located between N and C modulates solubility. The Sup35NM fragment is typically employed for studying aggregation *in vitro* and is sufficient to transmit all information required for prion proliferation when transformed into the yeast cell (Tanaka et al., 2004).

Yeast prions, and specifically Sup35 (or Sup35NM) protein, provide a tractable experimental tool to investigate the conditions for the heat inactivation of self-perpetuating protein aggregates. In this work, we employed Sup35NM aggregates as a biological indicator for inactivation of prion agents using the potential in-flight sterilization modalities dry heat.

## Materials and methods

### Expression and purification of Sup35NM-His_6_

*Escherichia coli* strain HMS174 (DE3) pLysS (Novagen) was transformed with pET21b vector containing the NM coding region of Sup35 from *S. cerevisiae* with an attached C-terminal His_6_ tag (Allen et al., 2005). Sup35NM-His_6_ was expressed and purified as described previously (Yeh et al., 2010). In brief, competent HMS174 (DE3) pLysS cells were transformed with the cloning vector. Sup35NM-His_6_ expression was induced using isopropyl *β*-D-1-thiogalactopyranoside at a final concentration of 1 mM and the cells were harvested following 4 h of induction at 37°C. Cell pellets were stored at-80°C prior to purification. Sup35NM-His_6_ was purified *via* Ni-NTA His-tag affinity purification under denaturing conditions. Sup35NM-His_6_ was precipitated in ice-cold methanol and the protein pellet was collected *via* centrifugation, washed with ice-cold methanol, and stored at-80°C in 80% methanol until use.

### Production of Sup35NM-His_6_ aggregates

Sup35NM-His_6_ stored in 80% methanol at −80°C was collected by centrifugation. The supernatant was discarded and the pellet was resuspended in 1XPBS. The concentration of protein was measured *via* Bradford’s Assay (BIO-RAD) and the solution diluted such that the concentration of Sup35NM-His_6_ was 1 mg/ml. The diluted protein solution was transferred to a U-bottom microcentrifuge tube and rotated end-over-end for 48 h at room temperature to form aggregates. Aggregate formation was checked using the “boiled gel” procedure and thioflavin T fluorescence (described below).

### Spotting Sup35NM-His_6_ aggregates on stainless steel coupons

After confirming aggregation, the concentration of the Sup35NM-His_6_ aggregates was adjusted to 1 mg/ml as per data by Bradford’s assay. Sterile stainless-steel coupons (Mesa Labs) with a total diameter of 9 mm were then unwrapped from autoclavable pouches and placed in a desiccator, and 50 μl of aggregate solution was pipetted onto each coupon, to cover the flat portion of a coupon with a diameter of 4.9 mm. Coupons were then placed under vacuum until completely dry. This was repeated twice to ensure a final protein amount of 0.1 mg per coupon.

### Exposure to TSEV

The prepared coupons were placed into individual Thermal Spore Exposure Vessels (TSEVs) or stainless-steel tubes and a temperature reference TSEV was exposed on each dry heat run cycle. An additional coupon was prepared (non-heat treated) to serve as a positive control. The TSEVS are placed into a constant high temperature silicone bath and the temperature of the bath was calibrated with a reference thermometer. The TSEVs are held in a modified lid of the bath and the thermocouple junction touched the bottom of the reference coupon to record the actual temperature to which the proteins were being exposed. The TSEV was immersed in the bath simultaneously and the time temperature of the reference TSEV was recorded every second (it takes 30–40 s for the TSEV to reach the set temperature). After heating, the TSEVs were removed from the oil bath and placed into an ice bath to cool. Resulting treated coupons were stored at −80°C and sent on dry ice from JPL where treatment was performed, to Georgia Tech for protein recovery and further analysis.

### Protein recovery from coupons

Using sterile forceps, coupons were lightly folded into a “U”-shape so that a coupon could fall into the bottom of a microcentrifuge tube. Once all coupons had been folded and placed into tubes, 200 μl of sterile 1X PBS was added to each coupon-containing tube. Coupons were vortexed for 1 hr then stored at −80°C until use.

### Detection of protein and aggregates

SDS-PAGE gel electrophoresis and Western blotting using His_6_ antibody (Abcam) were performed as described (Allen et al., 2005). Protein samples were combined in a 1:3 ratio with 4X Loading Buffer (240 mM Tris-Cl, pH 6.8, 8% SDS, 40% glycerol, 12% 2-mercaptoethanol, 0.002% bromophenol blue), either boiled for 10 min (in the case if aggregates would need to be solubilized) or not boiled (if aggregates were to be kept intact) and loaded on a 12% SDS-polyacrylamide gel with a 4% stacking gel. Electrophoresis was performed at approximately 100 V in Tris-glycine-SDS running buffer (25 mM Tris, 192 mM glycine, 0.1% SDS, pH 8.3). In the case of “boiled gel” assay for aggregate detection (Kushnirov et al., 2006), electrophoresis was halted after 1 h and the gel was removed from the running box. The wells were sealed with 4% acrylamide, allowed to polymerize, and the whole gel was wrapped in plastic wrapping and boiled for 10 min. After allowing several minutes for cooling, the gel was placed back in the cassette and electrophoresis continued for about 1.5 h. Proteins were transferred to Hybond-ECL nitrocellulose membrane (Amersham) using a Criterion™ blotter (BIO-RAD), and the membrane was blocked in 5% milk, and then incubated with His_6_ primary antibody (Abcam) followed by anti-Rb (HRP) secondary antibody (BioRad). Protein was visualized using ECL detection reagent (Amersham).

### Thioflavin T based seeding assay

Thioflavin T based fibrillization assays for detection of the amyloid-seeding activity were conducted as described previously (Sharma et al., 2018). Briefly, Sup35NM-His_6_ stored in 80% methanol at −80°C was collected by centrifugation. The supernatant was discarded and the pellet resuspended in 8 M urea buffer (8 M Urea, 100 mM NaH_2_PO_4_, 10 mM Tris, pH 8.0). Sup35NM-His_6_ was concentrated using a 3-kDa filter (EMD Millipore) and diluted 100-fold in 1XPBS. The protein concentration was determined *via* Bradford’s Assay (BIO-RAD). The Sup35NM-His_6_ monomers were boiled for 10 min prior to starting aggregation experiments to break down any pre-formed aggregates or oligomers.

A stock solution of 1 mM Thioflavin T (ThT) was prepared fresh in 1X PBS. Aggregation experiments were performed in triplicate in a black, clear-bottomed 96 well plate (Greiner CELLSTAR) with a final ThT, Sup35NM-His_6_ monomer, and Sup35NM-His_6_ aggregate concentration of 10, 5, and 0.025 μM, respectively. ThT assays were carried out in a SpectraMax iD3 microplate reader (Molecular Devices). Fluorescence was recorded every 10 min, with shaking in between readings, using an excitation wavelength of 440 nm and an emission wavelength of 486 nm. Fluorescent readings are interpreted as a measure of the total amount of amyloid aggregates formed.

## Results

### Recovery of Sup35NM-His_6_ aggregates after the dry heat treatment

We sought to develop a series of tests to access the retention and amyloid seeding activity of Sup35NM-His_6_ aggregates after exposures to dry heat for using it as a BI for sample return sterilization systems. Dry heat tests were performed at temperatures of 125, 250, and 350°C. To achieve this goal, the recombinant Sup35NM-His_6_ protein was purified from *E. coli*, aggregated and immobilized on stainless steel coupons, and subjected to temperature treatment as described in Materials and Methods. Protein recovered from coupons and stored in 1X PBS at −80°C was thawed at room temperature, and protein concentrations were determined using Bradford’s assay. Protein solution was then diluted, as appropriate, prior to loading onto the SDS-PAGE gel. Sup35NM-His_6_ aggregates produced *in vitro* were used as a loading control. Each protein sample was run on three gels: one gel to be stained with Coomassie Blue, and two gels that were transferred to nitrocellulose membrane and probed using anti-His_6_ antibody. Of these two gels, one was prepared using the “boiled gel” procedure, resulting in solubilization of aggregates at 1 h after the start of electrophoresis (see Materials and Methods). While aggregated protein cannot enter the gel, solubilized aggregates are converted into monomers entering the gel, so that in case of “boiled” gel, bands corresponding to initially monomeric protein and initially aggregated protein could be detected at different locations.

**Figure 1:**
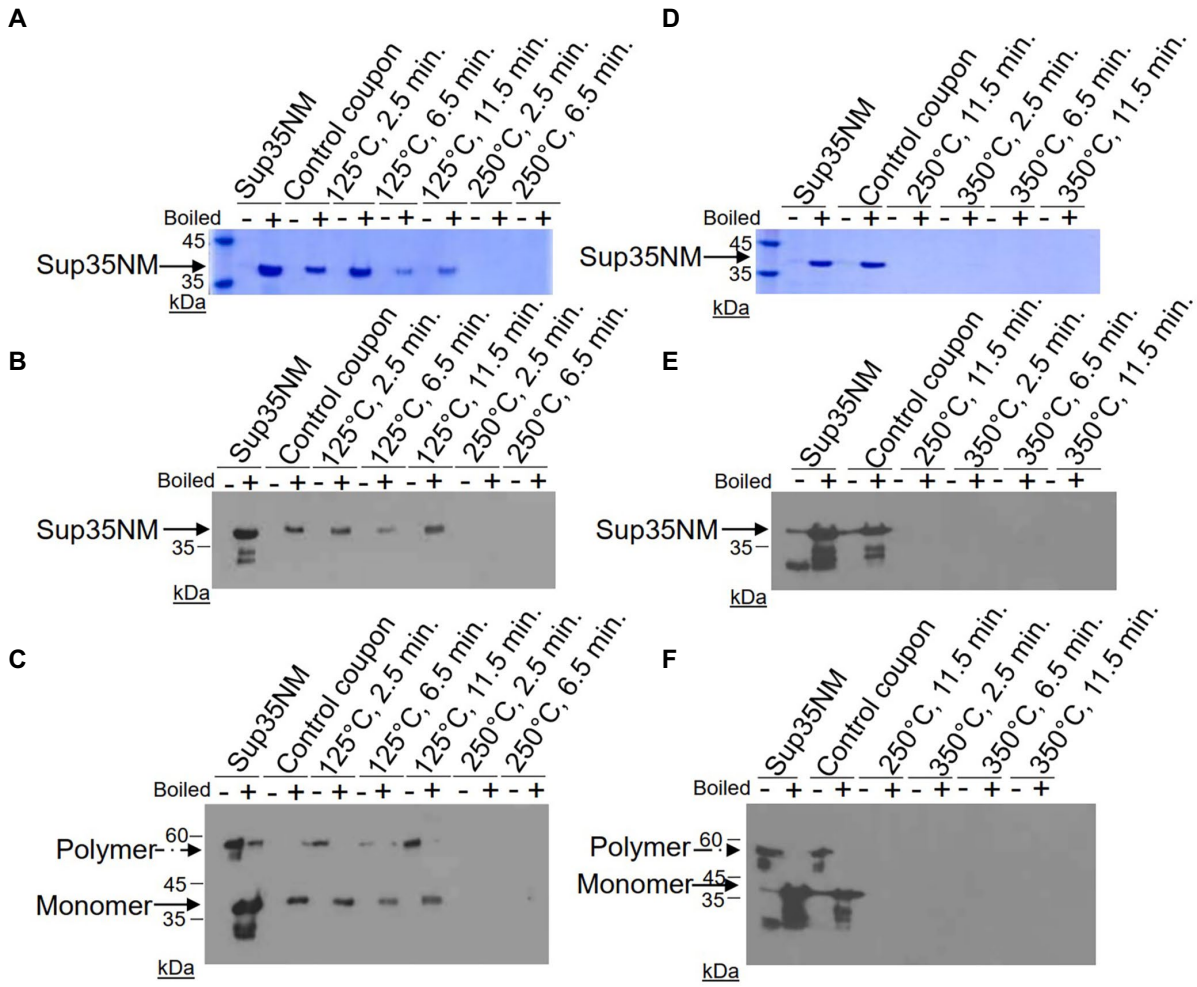
Detection of Sup35NM-His_6_ aggregates recovered from stainless-steel coupons by SDS-PAGE and Western blotting. Proteins from control coupons, and from coupons treated at 125°C, 250°C and 350°C for various periods of time were analyzed as indicated. *In vitro* aggregated Sup35NM not immobilized on a coupon was used as a control. Amount of proteins loaded was normalized according to Bradford assay. **(A,D)** SDS-PAGE gels stained with Coomassie Blue. Samples were alternated in such a way that not-boiled samples (−) were loaded first, followed by pre-boiled samples (+). Aggregated protein cannot enter the gel without pre-boiling. Left lane contains molecular weight markers. **(B,E)** SDS-PAGE gels analyzed by Western blotting, followed by reaction to anti-His_6_ antibodies. Sup35NM-Hi_s6_ monomer bands are indicated by the solid arrow. Lower bands likely represent proteolytic fragments. **(C,F)** Boiled SDS-PAGE gels analyzed by Western blotting, followed by reaction to anti-His_6_ antibodies. Aggregated protein (polymer) in non-boiled samples enters the gel after the gel is boiled. Positions of polymers and monomers are shown by arrows. Lower bands likely represent proteolytic fragments. Gels analyzed by Western were overexposed in order to see even residual amounts of immunoreactive material. Positions of nearest molecular weight markers are indicated on Western gels.

Results of these experiments are shown on Figure 1 and summarized in Table 1.* Despite some variability between experiments, protein recovery from control (untreated) coupons was reasonably good (in the range from 23% to 93%, see Table 1), and protein of expected size and aggregated state was always detectable by both Coomassie staining and Westerns (Figure 1; Table 1), indicating both that Sup35NM-His_6_ can bind stainless steel, as has been observed with PrP^Sc^ (Flechsig et al., 2001), and that Sup35NM-His_6_ aggregates are sufficiently stable to survive desiccation and storage. Protein recovered from coupons treated at 125°C for any period of time was also detectable by Bradford (Table 1) and SDS-PAGE (Figures 1A–C; Table 1). Amount of the Bradford-detectable material generally decreased (although remained significant, in the range from 7% to 23%) in solutions generated from coupons treated at 250°C or 350°C (Table 1). However, the protein of the expected size was neither seen on the Coomassie gel nor detected by Western (on both regular and “boiled” gels) after these treatments (Figure 1; Table 1). Thus, dry heat at high temperature results in significant degradation of Sup35NM.

**Table 1:** Summary of analysis of Sup35NM-His_6_ aggregates recovered from stainless coupons.

Treatment condition	Percent recovery (range)*	Detectability of protein by SDS-PAGE and Western	Seeding ability**
Control (None)	23–93	4 out of 4	4 out of 4
125°C, 2.5 min	47–48	3 out of 3	2 out of 2
125°C, 6.5 min	20–52	3 out of 3	2 out of 2
125°C, 11.5 min	28–70	3 out of 3	2 out of 2
250°C, 2.5 min	7–8	0 out of 3	0 out of 2
250°C, 6.5 min	15–18	0 out of 3	1 out of 2
250°C, 11.5 min	12–39	0 out of 6	0 out of 5
350°C, 2.5 min	10–25	0 out of 3	1 out of 2
350°C, 6.5 min	16–28	0 out of 3	1 out of 2
350°C, 11.5 min	4–23	0 out of 6	0 out of 5

* Determined by Bradford’s assay.

** Determined by ThT assay.

### Analysis of the amyloid seeding activity of Sup35NM-His_6_ after heat treatment

**Figure 2:**
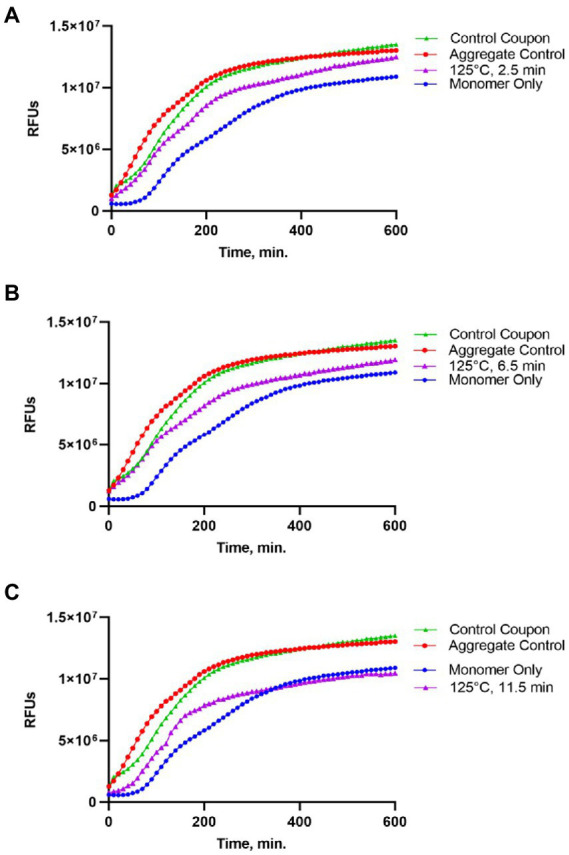
Impact of the 125°C treatment on the seeding activity of Sup35NM-His_6_ aggregates. Sup35NM-His_6_, recovered from coupons treated at 125°C for 2.5 min **(A)**, 6.5 min **(B)** and 11.5 min **(C)** was analyzed. The protein recovered from coupons (purple, empty triangles) was added to soluble Sup35NM-His_6_, monomers at 1:20 ratio according to Bradford. Unseeded monomer (blue, filled circles), and monomers seeded by *in vitro* obtained aggregates that were been immobilized on coupons (red, empty circles), or were recovered from untreated control coupons (green, filled triangles) were used for the comparison. Seeding activity is always detected after 125°C treatment, even though it is less efficient than by control aggregates. Typical examples are shown.

**Figure 3:**
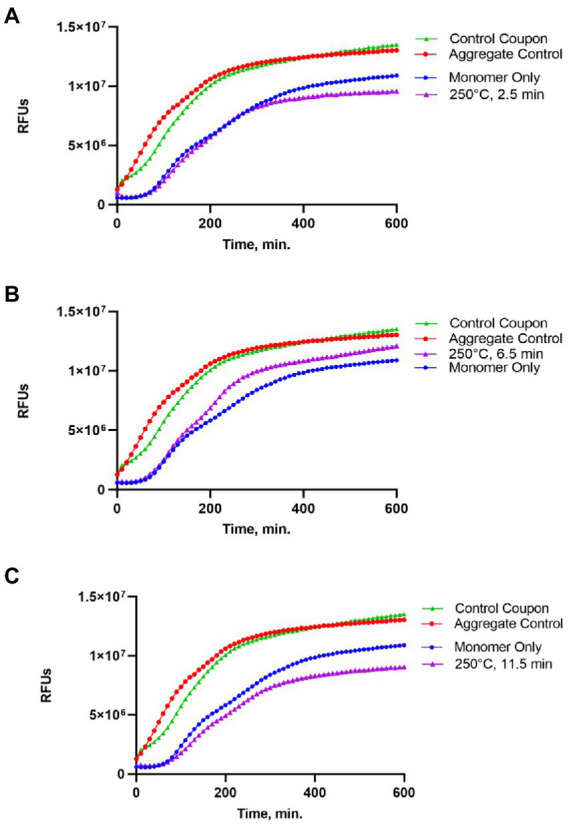
Impact of the 250°C treatment on the seeding activity of Sup35NM-His_6_ aggregates. Sup35NM-His_6_ recovered from coupons treated at 250°C for 2.5 min **(A)**, 6.5 min **(B)** and 11.5 min **(C)** was analyzed. Conditions of the seeding assay and designations are the same as on Figure 2. After 250°C treatment for 2.5 min (typical example is shown on **A**) or 11.5 min (typical example is shown on **C**), seeding activity was not detected, however it was detected in one of two samples treated for 6.5 min (shown on **B**).

**Figure 4:**
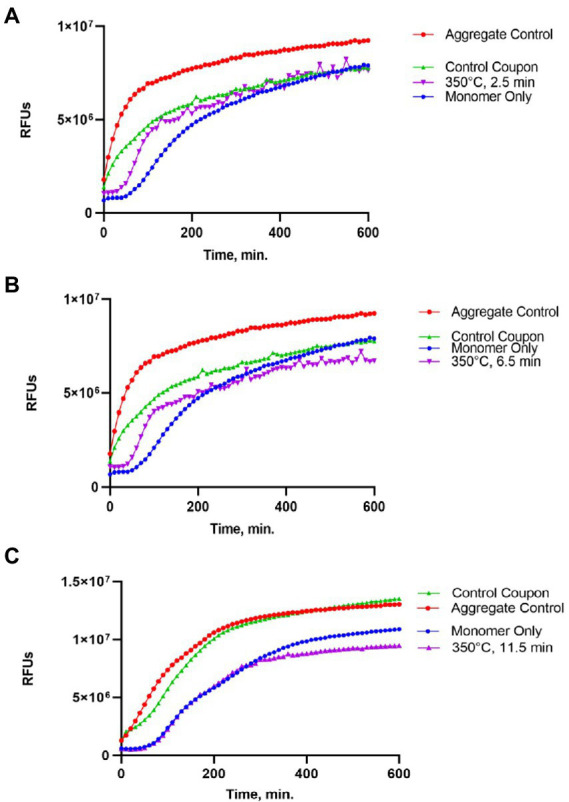
Impact of the 350°C treatment on the seeding activity of Sup35NM-His_6_ aggregates. Sup35NM-His_6_ recovered from coupons treated at 350°C for 2.5 min **(A)**, 6.5 min **(B)** and 11.5 min **(C)** was analyzed. Conditions of the seeding assay and designations are the same as on Figure 2. Weak seeding activity was detected in one out of two samples treated at 350°C for 2.5 min (shown on **A**), and in one out of two samples treated at 350°C for 6.5 min (shown on **B**), but it was not detected in any of 5 samples treated at 350°C for 11.5 min (a typical example is shown on **C**).

Following gel analysis, protein samples recovered from coupons were checked in the ThT based seeding assay (see Materials and Methods) to assess the propensity of recovered protein to seed Sup35NM-His_6_ aggregation. For this purpose, aggregated protein immobilized from coupons was added to soluble Sup35NM-His_6_ monomers in a ratio of 1:20, according to Bradford data. Aggregation of Sup35NM-His_6_ was detected by ThT binding, resulting in fluorescence as described in Materials and Methods. Recovered protein was considered capable of seeding if the aggregation lag time was shortened and the overall curve was shifted upwards compared to spontaneous aggregation of Sup35NM-His_6_ monomers. Recovered protein was considered inactivated if aggregation in the “seeded” sample occurred at a similar or reduced rate, compared to spontaneous aggregation of monomeric Sup35NM-His_6_. Results of these experiments are presented on Figures 2–4.

Protein recovered from control (not treated) coupons was always capable of seeding of Sup35NM-His_6_ monomers (see Control coupon curves on Figures 2–4), though somewhat less efficiently than the *in vitro* aggregated Sup35NM-His_6_ sample that has not been immobilized on a coupon and then recovered. This generally agreed with protein analysis data described above. Likewise, protein recovered from coupons treated at 125°C for any period of time was able to seed Sup35NM-His_6_ monomer aggregation, though less efficiently than protein recovered from control coupons or than the control Sup35NM-His_6_ aggregates (Figure 2; Table 1). In contrast, protein recovered from coupons treated at 250°C for 2.5 (Figure 3A) or 11.5 min (Figure 3C) was unable to seed Sup35NM-His_6_ monomer aggregation (see also Table 1). Interestingly, protein recovered from one of the two coupons treated at 250°C for 6.5 min was capable of seeding monomer aggregation, despite the lack of Coomassie staining and lack of immunoreactive material detectable by Western blot (Figure 3B; Table 1). Likewise, proteins recovered from one of the two coupons treated at 350°C for 2.5 min (Figure 4A; Table 1) and from one of two coupons treated at 350°C for 6.5 min (Figure 4B; Table 1) was capable of seeding aggregation, despite the lack of immunoreactive material. However, none of the five coupons treated at 350°C for 11.5 min produced any detectable seeding activity (Figure 4C; Table 1), confirming that this treatment consistently eliminates a biologically active prion.

## Discussion

### Dry heat as a tool for prion inactivation

Our data show that Sup35NM aggregates are reproducibly inactivated by the exposures to dry heat at 250°C or 350°C for 11.5 min. Shorter treatments at these temperatures led to inactivation of the seeding activity of Sup35NM in some but not all cases. It remains to be seen if these conditions are applicable to other proteins in prion form. Previous surface decontamination investigations reported that mammalian PrP retained functionality after exposure to 360°C for an hour and even following incineration at 600°C for 15 min (Brown et al., 2000), however another work indicated that dry-heating at temperatures of 600°C and higher (but not 400°C or lower) resulted in inactivation of the PrP-based prion agent of bovine spongiform encephalopathy (Matsuura et al., 2020). However, it is important to highlight that in these experiments, brain material was subjected to heat treatment. Therefore, it is possible that the heat was not evenly distributed throughout the material resulting in prion retention (Rohwer, 1984). Brain tissue is known to be hydrophobic with a high content of lipids and may have contributed to the “protective effect” where the tissue shields the protein from the sterilization source (Rohwer, 1984; Weinstein et al., 2001). For the application of our proposed tests, the surrogate prions were not embedded or prepared in brain homogenate consisted of purified surrogate prion proteins dessicated on a surface. Our approach appears to be more accurate for the development of space mission-related inactivation procedures. However, additional experiments with prion/amyloid proteins other than Sup35 are needed to determine the universality of a heat-based inactivation procedure.

### Mechanism of prion inactivation

To completely inactivate prions, they must be either denatured to the point where they are not able to refold into a prion shape, or completely physically destroyed. Prion protein renaturation has not been shown to occur once denaturation has been achieved (Prusiner et al., 1993). It is interesting that in our experiments, Sup35NM retained some seeding (prion) activity even in the conditions where no immunoreactive protein material was detected. One possibility is that while a significant portion of the protein was degraded in these conditions, a portion involved in an amyloid core remained. This assumption is logical as amyloid core is most resistant to damaging treatments. Indeed, our most sensitive antibodies were to the His_6_ tag, which was located at the C-terminus of Sup35NM fragment, that is, outside of the expected location of amyloid core. We also employed the polyclonal Sup35NM antibody (data not shown), but this antibody is much less sensitive, and is likely the least capable of recognizing the Sup35N region due to its poor immunogenicity. It remains to be determined if a complete loss of any detectable seeding activity at 350°C is due to complete destruction of the protein to short non-amyloidogenic peptides and/or to amino acids, or due to dissolution of remaining amyloid aggregates into non-prion monomers.

### Biological relevance of prions as indicators

As prions are completely dependent on a substrate protein produced by a host organism/cell for their reproduction, it seems most likely that any hypothetical Martian prion or similar protein assembly (if such an assembly exists and is present in the cached samples) would be incapable of propagating on Earth owing to the lack of available hosts capable of producing a substrate protein of the same or similar amino acid sequence. Also, protein folding and prion propagation depend on temperature and water availability. Since no liquid water has yet been found on Mars’ surface in our exploration to date, environmental conditions are not favorable for enabling the propagation of prions.

However, one should note that the recent evidence increasingly points to the ability of some prion and other amyloid proteins to cross-seed non-homologous substrates. Cross-seeding interactions have been described between protein as distant as yeast Sup35 and human tau (Flach et al., 2022) or bacterial curli and human synuclein (Sampson et al., 2020). Even though these cross-seeding interactions are less efficient than homologous seeding, the possibility of the existence of highly promiscuous prions with broad cross-seeding capabilities cannot be excluded. Therefore, further studies on prion inactivation remain relevant to the biosafety aspect of space missions.

## Data availability statement

The original contributions presented in the study are included in the article/supplementary material, further inquiries can be directed to the corresponding author.

## Author contributions

ES was the Science PI and directed experiments and wrote the manuscript draft. YC senior researcher directed the analytical experimentation and significantly improved this paper. AH carried out all of the laboratory analysis and produced all of the protein. WS PI carried out all of the heat inactivation experiments. All authors contributed to the article and approved the submitted version.

## Funding

Supported by the National Aeronautics and Space Administration: NNH18ZDA001N-PPR. The research described in this publication was carried out at the Jet Propulsion Laboratory, California Institute of Technology, under a contract with the National Aeronautics and Space Administration, Honeybee Robotics, and at Georgia Institute of technology under a subcontract from JPL.

## Conflict of interest

ES is employed by the company Honeybee Robotics.

The remaining authors declare that the research was conducted in the absence of any commercial or financial relationships that could be construed as a potential conflict of interest.

## Publisher’s note

All claims expressed in this article are solely those of the authors and do not necessarily represent those of their affiliated organizations, or those of the publisher, the editors and the reviewers. Any product that may be evaluated in this article, or claim that may be made by its manufacturer, is not guaranteed or endorsed by the publisher.
